# Irregularities

**Published:** 1887-05

**Authors:** J. E. Robinson


### Irregularities.

BY J. E. ROBINSON.

(Read Before the Cleveland Dental Society, March, 1887.)

### CAUSE AND TREATMENT.

Much has been written and considerable time given to discussions on the above subject during the past few years, and one can hardly hope to enlighten the members of this society to any great extent in the short time I shall claim your attention.

I shall touch but lightly upon, and lack of time may preclude any mention of, appliances or methods for "straightening" or returning teeth abnormally located to their normal position in the dental arch. In fact each individual case is so much of a study, and requires a treatment so exclusively its own, that without casts and drawings, or much greater skill at the blackboard than I possess, I should be unable to make myself clearly understood.

Many operators of good repute claim that nature, if undisturbed, will take care of the teeth in their growth and proper location, as well as all other physical developments, and that nothing further is required than to see that the deciduous teeth are retained to the utmost limit of possibility, in order to force a proper expansion of the arches of the mouth during the growth and eruption of the permanent or second dentition.

Some others, of no mean pretensions, have pronounced in favor of an early removal of the deciduous teeth, claiming that quite early in embryonic life the germs of some—and one quite eminent writer claims at birth as many as twenty-four—of the permanent teeth are located and ready for, and in process of, calcification; and if when developed and ready for eruption the absorption of the deciduous teeth is not delayed, thereby causing a struggle between them for position, there will be as few irregularities developed in the permanent as is found in the deciduous teeth. Their remedy is to watch closely, and if a lack of room presents itself, cause the removal of one or more of the permanent teeth—notably the "sixth year molars"—thereby gaining the needed room, and by frequent daily pressure on the part of the patient forward or backward, as the case may be, compel the advancing tooth to take its proper place in the arch.

While I can recognize in each of these treatments a course of procedure that is sometimes justifiable, I think we must as a rule, in all but the simplest cases, resort to surgical skill and mechanical force to assist nature to a proper development or arrangement. My reasons for this are that the causes of irregularities, or I might better say the cause, in a very large majority of cases, is of more remote origin than the simple premature or retarded eruption of a permanent tooth or teeth. We must study the laws of heredity if we would find the most prolific cause of irregularities. In all but the human animal care is taken that the physical characteristics of both father and mother are sufficiently in harmony to produce offspring well developed and of fair proportions, and with rare exceptions the end sought is attained.

With man little or no thought is given to posterity when choosing partners for life. We are guided by a sentiment called love, and, physically fit or unfit, we mate and reproduce our kind.

I have no quarrel with our method, indeed would not have it otherwise. Still the fact remains that the robust man, with massive jaw and teeth of corresponding proportions, quite often weds the woman of slight frame and slighter dental formation. In fact I believe we are oftenest charmed to wedlock by those of opposite physical and mental characteristics, and as a result a perfectly regular dentition is seldom produced. To be sure, in a great number of cases the deformity is not sufficient to need attention at our hands, and passes unnoticed. Nevertheless, I doubt if any dentist present has not many times in his practice been called upon to give advice, if not to treat, cases where the large teeth of one parent were encased in the small jaw of the other, and *vice versa.* In the first named we get the crowded condition to which our attention is most frequently called. When the inheritance is of the last mentioned kind we get the teeth with considerable space between, which is less unsightly, and less attention is given to them.

It may be that in other and older settled communities, where the population is not composed so largely of an admixture of races as is our own, this cause of irregularities is less frequently met. I apprehend that our brethren in the old world are much less frequently called upon to render professional services in this important and constantly growing branch of our practice.

The cause or causes just mentioned are to my mind predominant in producing oral deformities that require orthodontic treatment.

It has been suggested when informally conversing on this subject that the various eruptive fevers, so often occurring just prior to or during the period of second dentition, may, by retarding the growth of the jaw—the teeth having already assumed their form and size—be a prolific cause of irregularities. While I am not prepared to make a vigorous denial that such is the fact, I will say that so far as my own observation has extended I am unable to give the theory a full endorsement.

I hope, in the discussions that are to follow this paper, to hear this feature enlarged upon.

From the foregoing you will infer that my first action is to learn all that is possible of my patient's ancestry, both as regards form and nervous temperament; also to learn by close questioning—not alone of the patient and parents, but often of the family physician, who is in a better position than myself to judge—of the ability of the little one to pass safely through the ordeal, for 'tis sometimes better to " bear those ills we have, than fly to others that we know not of."

Some years ago a young miss, apparently in good health, was brought to the office of one of our leading dentists for the purpose of having some irregularities corrected. After thoroughly examining the case and watching closely the patient and some questioning, he became convinced that he saw symptoms of corea, and determined to learn the name of the family physician, to see if he also had detected any thing of the kind. He found that he was right in his conjecture, and concluded to wait awhile before operating. In a short time the disease developed, and partial paralysis of facial nerve became revealed, which may have been caused by some lesion of the portio dura, or by inheritance. Although this is an extreme case, and did not come under my own care, I mention it to show that too much care can not be taken to guard against like dangers. Had he operated as requested, without doubt the parents would mentally, if not loudly, have charged him with being the cause of the trouble.

Much diversity of opinion prevails as to the proper age to commence. My judgment is that, all other things being favorable, it is better to commence as young as possible, as then the bony structure yields more readily, the absorption under the stimulus of pressure is greater, and the filling up in the track of the moving teeth is more rapid.

Intermittent pressure is generally to be preferred to continuous, as we are much less liable to overdo when through failure of the patient to keep appointments the pressure is not removed or slackened at the proper time. Again, with intermittent pressure the severe pain soon subsides after the dismissal of the patient, and the operator is less liable to have his work undone or retarded through the mistaken kindness of the tender hearted parent or guardian, by removing the appliance to give relief. The getting the teeth in situation is a trifle slower, but we are compensated by a firmer condition when we are ready to place our retaining plate or band upon them, and no time is lost in fact.

Although a certain amount of invention is necessary in all cases, it is not as indispensable now in preparing appliances as formerly, as we have our Keely, Kinsgley, Coffin, Patrick, Farrar, Talbot, and others who have invented and given to the profession forms that with slight modifications and combinations one with the other, to suit the case in hand, almost any change in position can be accomplished. First study well our case, and after determining well our method of procedure, avail ourselves of these unpatented, freely-given, and, in too many instances, unacknowledged gifts of noble men, always bearing it in mind that the simpler the contrivance can be made and do its work the better the result, as thereby the liability to discouragement and perhaps abandonment is in a great measure prevented.

Although it was my intention to say nothing of my individual method of treatment, I must before closing impress upon your minds the necessity of having a perfect antagonizing model of the mouth before attempting any change. In the many cases to which my attention has been called by model but very few have paid any attention to getting any more than an impression of the teeth, and their articulation one jaw with the other.

Dr. Keely, of Oxford, who has made the correcting of irregularities almost a specialty of late, is particularly careful to obtain in addition to a good articulating model a perfect impression of the gum to the utmost limit as then not only the erupted teeth, but those in process of euption, can be considered. I find that so much more can be learned from a model taken as indicated that I make it conspicuous, even at the risk of repeating what may be known to all.

This paper has now progressed much further than I intended at the commencement, and I am just at the beginning of the subject. I leave it in your hands, hoping at some future day to continue to the end, and perhaps from time to time show you the progress of the, to me, interesting case I had the honor to present to your notice at our February meeting.

